# Antitumor effect of selenium-rich Brazil nuts and selenomethionine dietary supplementation on pre-existing 4T1 mammary tumor growth in mice

**DOI:** 10.1371/journal.pone.0278088

**Published:** 2023-01-12

**Authors:** Marina Apocalypse Nogueira Pereira, Ediu Carlos da Silva Junior, Istefani Luciene Dayse da Silva, Bárbara Andrade de Carvalho, Enio Ferreira, Eric Francelino Andrade, Luiz Roberto Guimarães Guilherme, Luciano José Pereira

**Affiliations:** 1 Department of Veterinary Sciences, Universidade Federal de Lavras (UFLA), Lavras, Minas Gerais, Brazil; 2 Department of Soil Sciences, Universidade Federal de Lavras (UFLA), Lavras, Minas Gerais, Brazil; 3 Department of Pathology, Universidade Federal Fluminense (UFF), Niterói, Rio de Janeiro, Brazil; 4 Biological Sciences Institute (ICB), Universidade Federal de Minas Gerais (UFMG), Belo Horizonte, Minas Gerais, Brazil; 5 Department of Health Sciences, Universidade Federal de Lavras (UFLA), Lavras, Minas Gerais, Brazil; University of Life Sciences in Lublin, POLAND

## Abstract

Selenium (Se) is an essential micronutrient known to play an important role in the antioxidant system that can potentially influence tumor growth. We aimed to investigate the effects of dietary Se supplementation after detection of 4T1 mammary tumor growth in *BALB/c* mice. Thirty female mice received subcutaneous inoculation of 4T1 cells. After five days, all animals presenting palpable tumors were randomly assigned to three groups: a control group (Se-control) receiving a diet with adequate Se (0.15 mg/kg) and two other groups that received Se-supplemented diets (1.4 mg/kg of total Se) with either Brazilian nuts (Se-Nuts) or selenomethionine (SeMet). Data were assessed by either One or Two-way ANOVA followed by Tukey’s HSD or Bonferroni’s post hoc tests, respectively. Both Se-supplemented diets reduced tumor volume from the thirteenth day of feeding compared with the Se-adequate (control) diet (p < 0.05). The SeMet group presented a higher Se blood concentration (p < 0.05) than the Se-control group, with the Se-Nuts group presenting intermediate values. Selenoprotein P gene expression in the liver was higher in the Se-Nuts group than in the Se-control group (p < 0.05), while the SeMet group presented intermediate expression. Dietary Se supplementation, starting after detection of 4T1 palpable lesions, reduced tumor volume in mice.

## Introduction

Cancer is a leading cause of death in countries of all income levels [1]. Breast cancer is considered the most prevalent type in women, representing almost 12% of all cancer cases of both genders and 6% of all deaths worldwide [2]. Breast cancer is generally recognized to be a multifactorial disease [3] in which oxidative stress has been related to a higher risk of cancer development and to faster neoplastic progression [4, 5]. The imbalance between antioxidant systems and the production of reactive oxygen species (ROS), such as superoxide (O_2_^-^) and hydrogen peroxide (H_2_O_2_), characterizes oxidative stress [6]. These molecules can damage DNA [7, 8] and lead to mutations in tumor-related genes [9, 10]. ROS present a paradox in their biological function in which low doses can modulate immune system playing an essential role in apoptosis, whereas higher doses lead to a disbalance in antioxidant status, favoring carcinogenesis [11, 12]. In higher concentration ROS can interact with surface and intracellular receptors, modulating signaling pathways, and disrupt physiological mechanisms related to proliferation, apoptosis and angiogenesis, that causes a pivotal step in carcinogenesis [13]. Thus, the role of ROS in cell proliferation may be mediated by direct or indirect activation of signaling pathways related to cell growth, such as p38MAPK, p70S6K and p90Rsk, JAK/STAT, phospholipase D, JNK and ERK [13, 14]. Furthermore, breast cells are damaged by ROS via estrogen induced oxidative stress in combination with receptor mediated proliferation of damaged cells [13]. This event causes an imbalance in cellular prooxidant/antioxidant status, which initiates breast cancer development [13]. Therefore, the concurrent use of antioxidants to control ROS formation has been proposed [15].

Selenium (Se) is an essential micronutrient [16, 17] known to play an important role in the antioxidant system [18, 19]. Despite some controversial epidemiological data [20], evidence suggests that inorganic and organic forms of Se affect cancer initiation and progression [21–23]. These protective effects, which include decreased mortality in cancer patients [23], have been associated with different mechanisms [24], especially selenoprotein glutathione peroxidase (GPx) [23, 25] (a family of antioxidant enzymes) [26], and are recognized for having protective effects against tumor development [27]. In addition, selenium inhibits the cytochrome P450 system Phase I enzymes, which normally convert chemical carcinogens into reactive DNA-attacking adducts, besides protecting DNA damage by increasing the activity of DNA repair enzymes, such as DNA glycosylases, and repair pathways that involve members, such as p53 and BRCA1 [28]. Furthermore, selenium compounds can inhibit estrogen receptor α (ERα) signaling in ER-positive MCF-7 breast cancer cells as evidenced by decreased estradiol-dependent cell growth and gene expression [29]. Selenium is also present in thioredoxin reductase (TrxR), another enzyme known to protect DNA and other cellular components against oxidative damage [30].

Dietary Se can be present in organic forms, such as selenocysteine (SeCys) and selenomethionine (SeMet), or inorganic forms, such as selenite (SeO(OH)_2_) and selenate (SeO_2_(OH)_2_) [31]. Natural sources include cereals, nuts, meat, some vegetables, and seafood. The amount of Se in food is highly variable and can be related to the Se content in the soil, in addition to other factors [32, 33]. Brazil nuts (*Bertholletia excelsa*) are known to have high levels of Se, varying from 0.2 to 512 mg Kg^-1^ [34, 35], with substantial bioavailability [36, 37]. Brazil nuts are known as one of the major food sources of selenium [38], and due to this, it is important to investigate the effects of supplementation with this compound in an experimental model of breast cancer.

Dietary Se supplementation has been associated with a reduced incidence of breast cancer [39]. However, the efficacy of Se supplementation depends on the dose and chemical form of Se [20] and the onset of administration: before tumor detection (preventive measure) or after tumor detection (adjunct therapy). However, the effects of Se supplementation after tumor detection have not yet been well elucidated in the literature, requiring further investigation. Distinct forms of Se at various concentrations can induce dramatically different biological effects [8, 40]. Although scientific reports have shown encouraging results with Se as a therapeutic agent *in vitro* [41], this response may depend on the stage of carcinogenesis in which administration begins [42, 43]. Thus, we aimed to evaluate the effects of dietary Se supplementation (SeMet and Brazilian nuts) on tumor growth, blood Se levels, hepatic GPx activity, SELENOP expression, and tumor and metastatic histomorphology in a 4T1 mouse breast cancer model starting consumption after tumor detection.

## Materials and methods

### Experimental animals

Seven-week-old female *BALB/c* mice (*Mus musculus*) weighing 20 to 24 g were provided by the Central Animal Bioterium of Universidade Federal de Lavras, Brazil. The animals were distributed into collective boxes (five animals per box) with dimensions of 410 x 340 x 175 mm. The room had a constant temperature of 25 ± 2°C and 12-hour light-dark cycles.

Previously, the animals underwent an adaptation period of seven days receiving deionized water and commercial feeding. Mice had food and water *ad libitum*, and their weight was determined periodically. This study was carried out in strict accordance with the recommendations in the Guide for the Care and Use of Laboratory Animals of the National Institutes of Health. The animal study was approved by the UFLA’s Ethical Committee in Animal Use (CEUA/UFLA) under protocol number 079/16. After the acclimation period, the animals were randomly divided into three groups (n = 7–9). A power calculation was performed to determine the sample size. The animal was considered the study unit. The sample size was determined to provide 80% power to recognize a significant difference of 20% among groups and a standard deviation of 15% with a 95% confidence interval (α = 0.05).

### Cell culture

The 4T1 cell line was obtained from the American Type Culture Collection (ATCC, USA) and was routinely cultured in Dulbecco’s modified Eagle’s medium (DMEM) supplemented with 1% penicillin/streptomycin and 10% fetal bovine serum (FBS; Thermo Fisher Scientific) at 37°C in a humidified 5% CO_2_ atmosphere. Cultures were tested for mycoplasma contamination by immunofluorescence staining with a 1 μg/mL solution of 4’,6-diamino-2-phenilindole (DAPI; Sigma-Aldrich).

### Experimental design

On the inoculation day, each animal received a subcutaneous injection of 0.1 mL containing 1 x 10^6^ tumoral cells diluted in phosphate buffered saline (PBS; Sigma). The solution was injected into the mouse flank, and tumors were measured daily until all animals presented palpable tumors.

**Fig 1:**
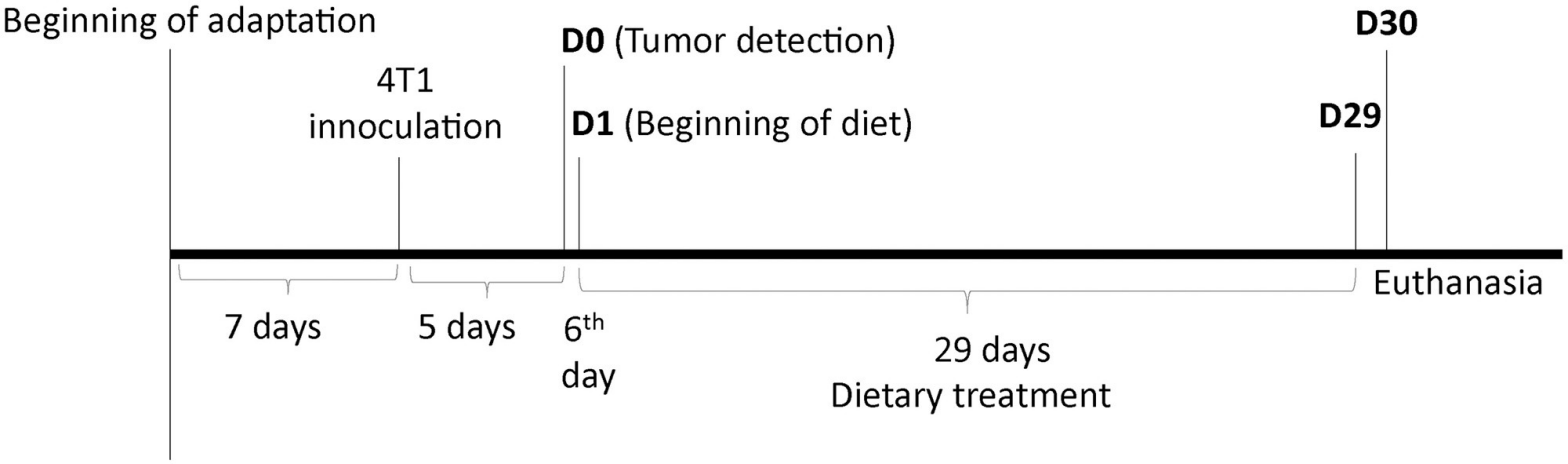
Fluxogram representing the experimental design over time. Animals started experimental diets six days after 4T1 inoculation to characterize a treatment study. After 29 complete feeding days all animals were euthanized for blood, tumor, liver and lung sampling/extraction.

All animals presented detectable tumors on the 5^th^ day after inoculation, and then, the tumor was measured every 48 hours (see below). On the sixth day, the animals were randomly distributed into one of three experimental diets (Se-adequate–control group; Se-supplemented–Se-Met; Se-Nut–Brazil nut), as shown in Fig 1.

### Measurement of Tumor Volume

Tumor diameters were measured using a caliper (Western, measurement 0.05 mm, ref. 1944). Cumulative tumor volume was estimated according to previous studies [44, 45] using the following formula: tumor volume = (length x width^2^)/2, in which “length” is the longer diameter and “width” is the shorter diameter. Cumulative tumor volume along the experiment was calculated based on four measurements over a total of 29 days, as described further.

The first tumor measurement occurred on D0, which was five days after 4T1 cell inoculation and one day before initiating the experimental diets (D1). The animals were randomly distributed into three treatment groups with similar initial tumor volumes (*p* > 0.05). Cumulative tumor volume was compared among the groups each two days along the experimental period (29 days–from D1 to D29) considering the interaction time*treatment (diet).

### Experimental diets

Mice received experimental diets for 29 complete days, with Se-adequate (0.15 ppm total Se) or Se-supplemented (1.4 mg kg^-1^ total Se) levels. Adequate levels were based on AIN-93 M feed for laboratory rodents, and supplementary levels were based on previous reports [45, 46]. The total Se amount in the diets was analytically determined by atomic absorption spectrometry with a graphite furnace (GF-AAS) [33]. The Se-adequate diet contained sodium selenate, while the Se-supplemented diets contained either SeMet (Selisseo 2%, Adisseo^®^, Brazil) or Brazil nuts (*B*. *excelsa*) provided by Aruanã Farm (Itacoatira, AM, Brazil) (Table 1).

**Table 1:** Ingredients of the experimental diets.

	Se-adequate (0.15 mg kg^-1^ Se)	Se-supplemented (1.4 mg kg^-1^ Se)	
	Sodium selenate	SeMet	Se-Nuts
**Starch (g)**	609.8	609.8	609.8
**Casein (g)**	200	200	197.5
**Cellulose (g)**	50	50	49.2
**Sucrose (g)**	50	50	48
**Soybean oil (mL)**	40	40	30.5
AIN-93 M Mineral Mix*^1^ **(g)**	35	35	35
AIN-93 M Vitamin Mix*^2^ **(g)**	10	10	10
**Methionine (g)**	3	3	3
**Choline (g)**	2	2	2
**BHT (g)**	0.2	0.2	0.2
**Selisseo**^**®**^ **2% Se (g)**	-	0.0035	-
**Brazil nut (g)**	-	-	14.8
**kcal/kg diet**	3759.2	3759.2	3753.8

*^1^ Mix by Rhoster^®^ Co. Mineral element content (g/kg mix): 357 g of calcium carbonate, anhydrous (40.04% Ca); 250 g of potassium phosphate, monobasic (22.76% P; 28.73% K); 209.806 g of powdered sucrose; 74 g of sodium chloride (39.34% Na; 60.66% Cl); 46.6 g of potassium sulfate (44.87% K; 18.39% S); 28 g of potassium citrate, tri-potassium, monohydrate (36.16% K); 24 g of magnesium oxide (60.32% Mg); 6.06 g of ferric citrate (16.5% Fe); 1.65 g of zinc carbonate (52.14% Zn); 1.45 g of sodium meta-silicate, 9 hydrate (9.88% Si); 0.63 g of manganous carbonate (47.79% Mn); 0.30 g of cupric carbonate (57.47% Cu); 0.275 g of chromium potassium sulfate, 12 hydrate (10.42% Cr); 0.0815 g of boric acid (17.5% B); 0.0635 g of sodium fluoride (45.24% F); 0.0318 g of nickel carbonate (45% Ni); 0.0174 g of lithium chloride (16.38% Li); 0.01025 g of sodium selenate, anhydrous (41.79% Se); 0.01 g of potassium iodate (59.3% I); 0.00795 g of ammonium paramolybdate, 4 hydrate (4.34% Mo); 0.0066 g of ammonium vanadate (43.55% V).

*^2^ Mix by Rhoster^®^ Co. Vitamin content (g/kg mix): 974.655 g of powdered sucrose; 15 g of vitamin E (all-*rac*-a-tocopheryl acetate) (500 IU/g); 3 g of nicotinic acid; 2.5 g of vitamin B-12 (cyanocobalamin) (0.1% in mannitol); 1.6 g of Ca pantothenate; 0.8 g of vitamin A (all-*trans*-retinyl palmitate) (500,000 IU/g); 0.7 g of pyridoxine-HCl; 0.6 g of thiamin-HCl; 0.6 g of riboflavin; 0.25 g of vitamin D_3_ (cholecalciferol) (400,000 IU/g); 0.2 g of folic acid; 0.075 g of vitamin K (phylloquinone); 0.020 g of D-biotin.

The Se content and centesimal composition of Brazil nut samples were analyzed to calculate the necessary amount of material to reach 1.4 mg kg^-1^ in the diet. Experimental diets were balanced with similar calorie and macronutrient proportions. Nuts were evaluated for dry matter, humidity, oil, protein, mineral residues and crude fiber according to AOAC (2005) [47] and were also submitted to α and γ tocopherol quantifications.

For Se content analysis of the Brazil nuts, five small paper bags containing four nuts each were dried using an oven at 60°C until they reached a constant weight (~72 h). Then, the samples were shelled and ground with a portable electrical mill (A11 basic analytical mill, IKA^®^, Staufen, Germany). The digestion process started when the samples in glass tubes received 6 mL of nitroperchloric acid at a proportion of 2:1 (v/v). Extracts were left overnight (~12 h), and then, the batch was digested.

The digestion procedure was initiated at 50°C and increased 50 ºC every 30 minutes until 200°C was reached. Analytical determination of total Se in the samples was performed using an atomic absorption spectrophotometer with a graphite furnace (GF-AAS). For quality control, for each batch, a standard reference material (White Clover—BCR 402, Institute for Reference Materials and Measurements, Geel, Belgium) containing 6.70 mg kg^-1^ Se was included. The average recovery rate for the SRM (*n* = 2) was 78.38%.

Selisseo 2% Se (Adisseo^®^, Brazil) was added to the SeMet diet as a source of hydroxy-SeMet (CH_3_Se-(CH_2_)_2_-CH(OH)-COOH). It is a white powder containing 5% of SeMet. Diets were mixed in special bowls previously cleaned with 10% nitric acid solution (HNO_3_), and staff manipulated all ingredients with nitrile gloves.

### Blood, tumor, liver and pulmonary sampling, preparation, storage and analysis

After twenty-nine complete days of receiving experimental diets, and fasting for eight hours, all animals were anesthetized using intraperitoneal injections of ketamine (90 mg kg^-1^) and thiopental sodium (60 mg kg^-1^) and then euthanized by cervical displacement. None of the animals was excluded throughout the experiment. All materials were manipulated with nitrile gloves previously washed with 10% nitric acid cleaning solution (HNO_3_).

Blood was collected by intracardiac puncture and immediately stored on ice using heparinized tubes. Samples were analyzed by the Chemical Analyzes Laboratory (LACHEM, RS, Brazil) for whole blood total Se quantification. The samples were digested through the acid method (USEPA 3050B) and submitted to hydride generation atomic absorption spectrometry (HG-AAS), as described by Olson et al. [48].

The primary tumor, liver and the right lung from each animal were collected for metastatic histological analysis. The right lung originated from three samples from different anatomic areas, called the “cranial”, “medial” and “caudal” lobes. Tissues were fixed in 10% buffered formalin solution and processed using the routine paraffin inclusion technique. Histologic sections (4 μm) were stained with hematoxylin-eosin techniques for morphologic and morphometric assessments [49]. Liver samples were also immediately stored in liquid nitrogen and stored in a -80°C ultra-freezer for further enzymatic and molecular analyses (described below).

### Measurement of hepatic GPx-1 activity

After euthanasia, hepatic samples were homogenized in 1 mL of cold buffer (50 mM Tris-HCl, pH 7.5, 5 mM EDTA and 1 mM DTT) per 100 milligrams of tissue with 3 cycles of 10 seconds at 13,000 rpm (T 25 basic Ultra-Turrax, IKA^®^, Staufen, Germany). Homogenates were then centrifuged at 17.760 x g for 15 minutes at 4°C to collect the supernatant, and assay samples were kept on ice in accordance with the manufacturer’s protocol (Glutathione Peroxidase Assay Kit, Cayman Chemical Company^®^, Ann Arbor, USA). Enzymatic activity was measured every 30 seconds for 6 minutes using a microplate reader with absorbance at 340 nm.

Protein concentrations were measured by an adaptation of the Bradford assay [50]. As a standard, bovine serum albumin (BSA; Sigma^®^) was diluted to 10 different concentrations from 0.3 mg/mL to 3 mg/mL, while samples were diluted at 1:100. Then, 125 μL of Bradford Reagent (Bio-Rad Protein Assay Dye Reagent Concentrate, Bio-Rad^®^, Cat #5000006) was added to every 25 μL of sample and incubated with gentle shaking for 5 minutes. Microplates were assessed for absorbance at 595 nm using a spectrometer and Gen5 Software.

### Analysis of SelP expression by real-time PCR

Hepatic samples were homogenized (T 25 basic Ultra-Turrax, IKA^®^, Staufen, Germany) with 1 mL of TRIzol Reagent (Thermo Fisher Scientific^®^). The interphase was collected after centrifugation at 12,000 x g for 10 minutes at 4°C and then mixed with 200 μL of cold chloroform for 40 seconds. The samples were left at room temperature for 10 minutes and then centrifuged at 12,000 x g for 15 minutes at 4°C. The aqueous phase was collected, gently mixed with 500 μL of isopropanol and left on ice for 10 minutes for further centrifugation at 12,000 x g for 10 minutes at 4°C. The precipitate was collected, shaken with 1 mL of 75% ethanol, and centrifuged at 12,000 x g for 10 minutes at 4°C. Resuspension was performed by mixing 40 μL of Milli-Q water and was kept refrigerated for RNA quantification in a nanospectrophotometer (NanoDrop^®^). The integrity of the RNA samples was verified by agarose gel electrophoresis and spectrophotometry. No signs of degradation were observed, and the absorbance values were nearly 2.0 at 260/230 and 260/280 nm. cDNA was synthesized using an iScript^™^ cDNA Synthesis Kit (Bio-Rad) with 1 μg of RNA, according to the manufacturer’s instructions. RT-qPCR was performed on a Rotor-Gene Q (Qiagen) apparatus using a QuantiNovaTM SYBR^®^ Green PCR kit (Qiagen) with 7.5 μl of SYBR, 3 μl of each primer (2 μM final concentration), and 1.5 μl of cDNA for each reaction.

The primers targeted an exon-exon region, and the sequences of the oligonucleotides were as follows: reference genes GAPDH (fw ACGGCCGCATCTTCTTGTGCA) and (rw CGCCCAAATCCGTTCACACCG) and SELENOP (fw TGTTACAAAGCCCCGGAGTG) and (rw GGTCTTCCAATCTGGATGCCTG). The diets were analyzed in technical duplicates for each biological triplicate. The expression analyses were performed using the ddCT method, and the means were normalized relative to the lowest treatment value for each gene.

### Primary tumor, liver, and lung histology

We evaluated the percentages of neoplasia, necrosis, inflammation, hemorrhage, and normal tissue areas in histologic tumor sections. Moreover, the mean diameters of nucleus were obtained by measuring the longitudinal and transverse diameters of 30 neoplastic cell nuclei. A correction factor obtained by a micrometer slide scale was employed for the means. Histological images were captured using a digital Spot Insight Color camera adapted to an Olympus BX-40 microscope. Tumor cell proliferative activity was evaluated by the mitotic index in 10 fields with a 40x objective. SPOT^®^ software version 3.4.5 and Corel DRAW^®^ software version 7.468 was used for image analysis.

In histologic lung sections, the presence or absence of metastasis was recorded, as well as the metastatic pattern (unique node or multiple nodes). Additionally, the intratumoral inflammatory infiltrate was evaluated considering two factors: intensity (discrete, light, moderate or heavy) and profile (mononuclear, polymorphonuclear or mixed–mononuclear and polymorphonuclear). All analyses were blindly performed by trained evaluators

### Statistical analysis

Data on total Se blood concentration, hepatic GPx activity, SELENOP expression and total tumor growth were submitted to analysis of variance (one-way ANOVA) [51], and when significant, means were compared among treatment groups using Tukey’s HSD test. Cumulative tumor volume was analyzed over time by two-way ANOVA followed by Bonferroni’s *post hoc* test (time*treatment/diet). We used the package Emmeans v2.23 [52] in R 3.4.4 [53] and GraphPad Prism software (version 5.01, GraphPad Software, San Diego, USA).

The Pearson correlation coefficient was calculated for blood Se concentration, GPx activity, SelP expression, total tumor growth at 28 days (from D1 to D29), tumor histomorphology and histomorphometry, and lung metastasis histologic characteristics.

## Results

### Selenium concentration and centesimal composition of Brazilian nuts

The mean Se concentration in Brazil nuts was 95.403 mg/kg. The centesimal composition of the analyzed Brazil nuts is shown below in Table 2. Values for α and γ tocopherols are expressed in mg tocopherol 100 g^-1^ of nut oil. The average level for α-tocopherol was 0.038 ± 0.007 mg, while for γ-tocopherol, it was 0.320 ± 0.010 mg.

**Table 2:** Centesimal composition of Brazil nuts (Aruanã Farm Itacoatira, AM, Brazil).

Sample	Dry Matter (%)	Humidity (%)	Fat (%)	Protein (%)	Mineral Residue (%)	Crude Fiber (%)
R1	98.69	1.31	58.68	17.60	3.44	5.27
R2	98.78	1.22	61.03	16.39	3.04	5.04
R3	98.80	1.20	58.70	17.44	3.55	5.43
**Mean**	98.76	1.24	59.47	17.14	3.34	5.25

Samples of Brazil nuts were divided into triplicates (R1, R2 and R3) for evaluation of dry matter, humidity, fat, protein, mineral residue, and crude fiber.

### Effects of dietary Se on tumor volume

**Fig 2:**
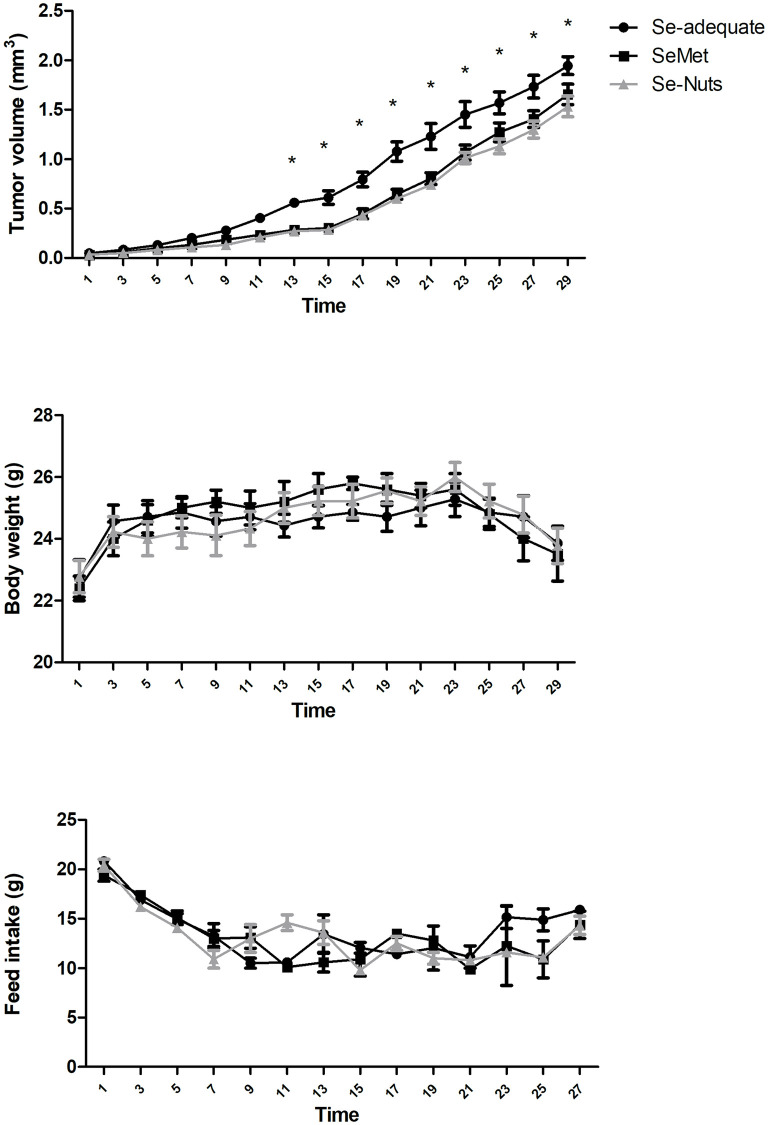
Tumor volume (mean ± SEM), body weight (mean ± SEM) and feed intake (mean ± SEM) over 28 days (from D1 to D29) in three experimental groups: Se-adequate (0.15 mg/kg total Se) and Se-supplemented diets (1.4 mg/kg total Se): SeMet and Se-Nuts (*n* = 7–9 for each group). *Statistically different by the Bonferroni’ test at *p* < 0.05.

All animals had palpable tumors by the 5^th^ day after 4T1 inoculation. Diets were initiated on the 6^th^ day (D1). Tumor volume did not differ among the three groups between D1 to D11 (*p > 0*.*05*). However, tumor volume became significantly lower in the Se-supplemented groups (SeMet and Se-Nuts) in comparison with the Se-adequate diet group from the 13^th^ day until the end of experiment (*p* < 0.05—Fig 2).

### Effects of dietary Selenium on the blood Se concentration, hepatic SelP expression, and GPx-1 activity

**Fig 3:**
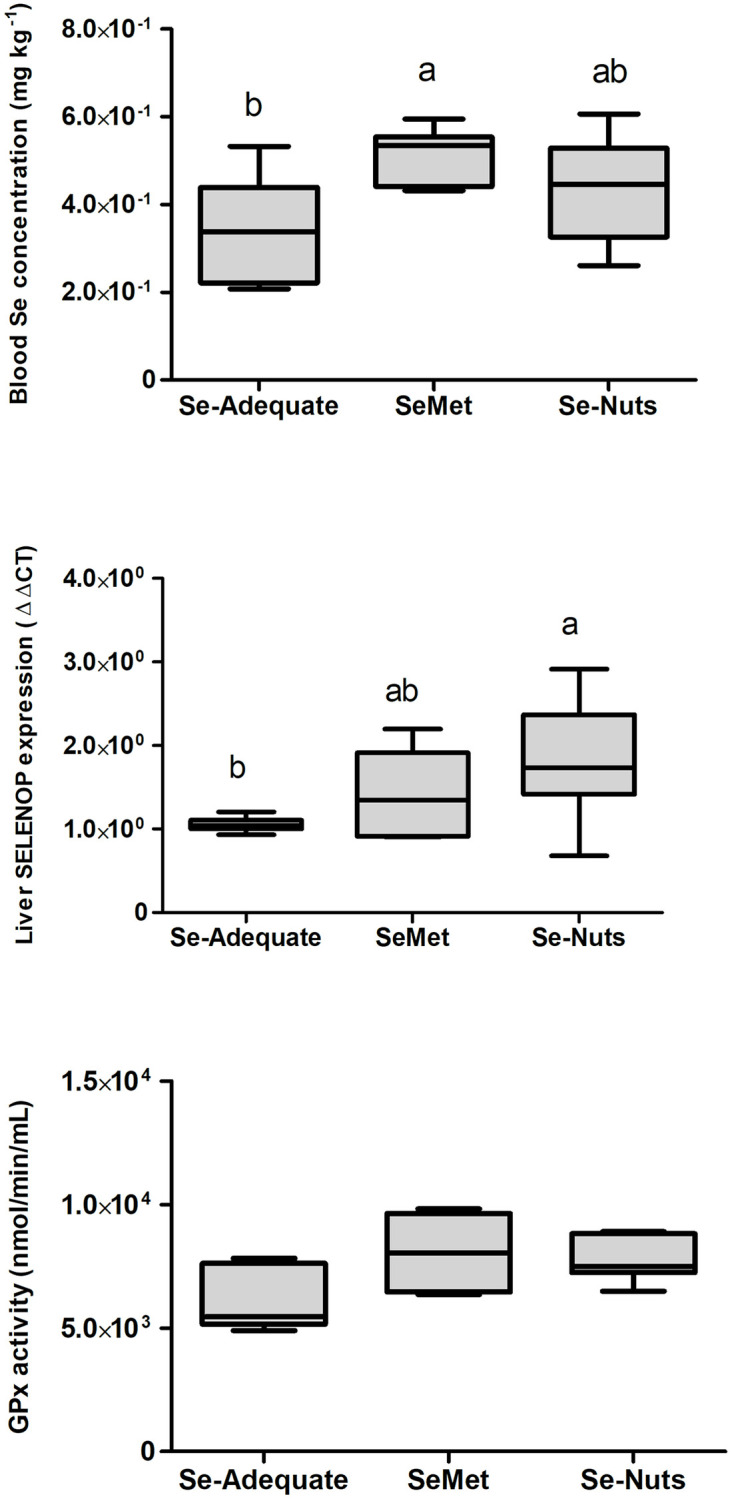
Mean (± SEM) blood Se concentration (μg mL^-1^). Hepatic SELENOP expression by real-time PCR and hepatic GPx-1 activity among different dietary groups: Se-adequate (0.15 mg kg^-1^ total Se), SeMet (1.4 mg kg^-1^ total Se) and Se-Nuts (1.4 mg kg^-1^ total Se) (*n* = 7–9). Average values followed by the same letters do not differ statistically by the Tukey test at *p* < 0.05 (a = a; a≠b).

A significant difference (p < 0.05) in blood Se concentration was observed only between the Se-adequate (0.15 mg/kg Se) and SeMet (1.4 mg/kg Se) groups (Fig 3). Hepatic SelP expression was higher (p < 0.05) in the Se-Nuts (1.4 mg/kg Se) group than in the Se-adequate group but did not differ from that in the SeMet group, which had an intermediate level (Fig 3). No significant differences were observed among the experimental groups in hepatic GPx-1 activity (Fig 3). There were significant negative correlations among blood Se concentration, GPx activity in the liver, and tumor volume at 28 days (Table 3).

**Table 3:** Pearson correlation analysis among blood Se concentration, GPx activity in liver, real-time PCR SELENOP expression and tumoral growth at 28 days.

Variables		*r*	*p*
Blood Se concentration	GPx activity	0.126	0.606
Blood Se concentration	PCR SELENOP expression	-0.345	0.147
GPx activity	PCR SELENOP expression	0.172	0.480
Blood Se concentration	Tumoral growth (28 days)	0.129	0.599
GPx activity	Tumoral growth (28 days)	-0.316	0.187
PCR SELENOP expression	Tumoral growth (28 days)	-0.374	0.115

### Effects of dietary Se on tumor histomorphology and metastatic histomorphology

**Fig 4:**
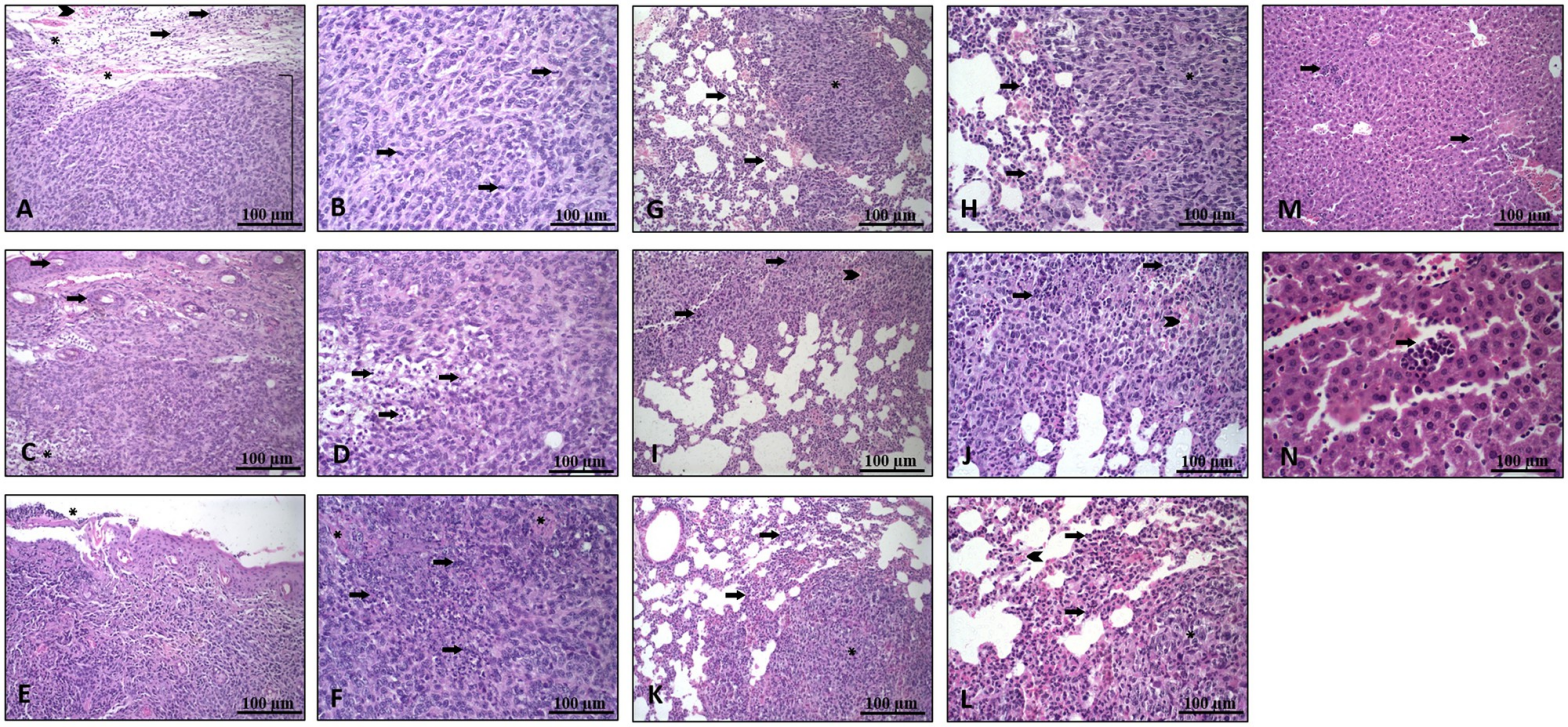
(A-F) Main tumor images: (A) Image (20x) showing a tumor area (bracket), with inflammation (arrows), peritumoral hyperemia (asterisks) and mild hemorrhage (arrowhead). (B) Greater magnification (40x) showing tumor cells, with apparent mitosis (arrows). (C) Image (20x) showing tumor cells compromising the upper and deep dermis with a focus of necrosis (asterisks), epidermis (arrow) and hair follicles. (D) Greater magnification (40x) showing an area of necrosis (cell debris and cells with a nucleus in pycnosis) (arrow). (E) Tumor reaching the epidermis (20x) and presenting an ulcerated area (discontinuity of the epithelium) (asterisk). (F) Tumor area (40x) showing intense necrosis (arrows) and hemorrhage (asterisks). (G-L) Lung images: (G) Lung fragment (20x) with metastatic focus (asterisk) and peritumoral polymorphonuclear inflammatory infiltrate (arrows). (H) Image showing (40x) the pattern of tumor cells (asterisk) and peritumor inflammatory cells (arrows). (I) Lung fragment (20x) with metastasis focus with moderate area of necrosis (arrows) and discrete hemorrhage foci (arrowhead). (J) Greater magnification (40x) showing an area of necrosis (arrows) and hemorrhage foci (arrowhead). (K) Lung fragment (20x) with metastatic focus (asterisk) and polymorphonuclear inflammatory infiltrate (arrows). (L) Image (40x) showing the pattern of tumor cells (asterisk), polymorphonuclear inflammatory cells (arrow) and hyperemia (arrowhead). (M-N) Liver images: (M) Liver fragment (20x) showing small foci of metastatic cells (arrow). (N) Greater magnification showing small foci of tumor cells (arrow).

The main tumor characteristics were similar among the groups regarding mitosis, nucleus diameter, and proportions of hemorrhage, inflammation, neoplasia, and necrosis (*p* > 0.05, Table 4; Fig 4A–4F). The pulmonary (Table 5; Fig 4G–4L) and hepatic (Fig 4M and 4N) metastatic characteristics were also similar regardless of dietary treatment.

**Table 4:** Histopathological characteristics of the main tumor.

	Se-Adequate	SeMet	Se-Nuts	p-value
**Mitosis (%)**	72.6	67.6	66.9	0.711
**Nuclear diameter (μm)**	8.17	7.97	8.29	0.363
**Hemorrhage (%)**	2.23	2.91	2.7	0.786
**Inflammation (%)**	15.1	16.8	18.3	0.666
**Neoplasia (%)**	57.5	58.1	58.4	0.995
**Necrosis (%)**	20.4	22.2	21.2	0.828

(n = 7-9/Group). No significant difference.

**Table 5:** Pulmonary metastatic characteristics (mean ± standard error) after 4T1 inoculation and 28 days of dietary treatment with Se-adequate (0.15 mg/kg total Se) and Se-supplemented diets (1.4 mg/kg total Se), which included SeMet or Se-Nuts.

	Se-Adequate	SeMet	Se-Nuts	P value
**Positive metastasis (%)** ^+^				
**Cranial lung lobe (%)**	80 (0.18)	83.3 (0.15)	42 (0.19)	0.229
**Medial lung lobe (%)**	66.6 (0.19)	40 (0.22)	75 (0.15)	0.439
**Caudal lung lobe (%)**	50 (0.20)	83.3 (0.15)	87.5 (0.12)	0.254
**Total (%)**	64.7 (0.12)	70.5 (0.11)	69.5 (0.10)	0.924
**Metastatic pattern (% of multiple nodules)** ^+^				
**Cranial lung lobe (%)**	50 (0.25)	60 (0.22)	66.6 (0.27)	0.902
**Medial lung lobe (%)**	50 (0.25)	50 (0.35)	50 (0.20)	1.000
**Caudal lung lobe (%)**	66.6 (0.27)	40 (0.22)	28.5 (0.17)	0.531
**Mean (%)**	54.5 (0.15)	50 (0.14)	43.75 (0.12)	0.854
**Inflammatory infiltrate intensity (1 = absent; 2 = mild; 3 = moderate; 4 = intense)** *				
**Cranial lung lobe**	3.60 (0.25)	3.33 (0.21)	3.42 (0.30)	0.724
**Medial lung lobe**	3.16 (0.40)	3.60 (0.25)	3.00 (0.27)	0.408
**Caudal lung lobe**	2.66 (0.33)	3.00 (0.37)	2.87 (0.35)	0.806
**Mean**	3.11 (0.21)	3.29 (0.17)	3.045 (0.18)	0.665
**Inflammatory infiltrate intensity pattern (1 = mononuclear; 2 = polymorphonuclear; 3 = mixed)** *				
**Cranial lung lobe**	1.80 (0.20)	2.16 (0.17)	1.85 (0.26)	0.452
**Medial lung lobe**	2.16 (0.31)	2.20 (0.20)	2.00 (0.19)	0.779
**Caudal lung lobe**	2.33 (0.33)	2.33 (0.21)	2.00 (0.27)	0.593
**Mean**	2.11 (0.17)	2.23 (0.11)	1.95 (0.13)	0.369

(n = 7-9/group);

^+^Chi-square test;

* Kruskal-Wallis test

## Discussion

The present study aimed to investigate whether dietary Se supplementation affected 4T1 tumoral volume associated with blood Se concentration, hepatic GPx-1 activity and SelP expression, and tumor and metastatic histomorphology. We observed that from the 13^th^ day of dietary treatment, cumulative tumor volume was significantly lower in both Se-supplemented groups (SeMet and Se-Nuts) than in the Se-adequate (control group). Tumor growth inhibition provided by SeMet can be due to SelP antioxidant activity in the plasma [54–56] since this group presented the highest blood Se concentration [57]. Other possible mechanisms previously associated with the anticancer effects of selenium include modulation of the p53 tumor-suppressor protein [58], SBP1 [59, 60], plasma GPx activity [61], Wnt signaling [62], induction of cancer cell apoptosis [63] and inhibition of tumor angiogenesis [64].

In human plasma samples, approximately 60% of Se is found as SelP and 3% in the form of GPx-3, with differences in these values compared to those in other animal species [65]. In whole blood, in addition to these selenoproteins, GPx-1 is deposited in erythrocytes and at lower concentrations in the form of SeMet, trimethylselenonium ions and selenosugar in red blood cells [66]. However, the Se forms found in blood may depend on the Se dietary source [67]. Women who ingested SeMet in a supplemental dose presented most of the blood Se in Hb, while blood Se was equally distributed between GPx and Hb in women ingesting selenate [68]. Similarly, rats fed selenite or SeCys had the majority of erythrocyte Se in the form of GPx, while mice that ingested SeMet, yeast or wheat had more Se deposited in hemoglobin (Hb) than in GPx [69]. In addition, Se from SeMet, unlike selenate and SeCys, can be incorporated into albumin [70]. Thus, the evaluation of the specific effect of the different sources of Se is necessary to understand which selenoprotein(s) show the greatest stimulation in each situation. For analysis of the status of Se, there is no single test, and the combination of several techniques is ideal [71]. The SelP concentration reflects the short-term status [72], as well as the level of Se in plasma or serum.

The smaller tumor volume observed in the SeMet group corroborates with reports by Chen et al. [45], who showed, through all 15 days of measurement, significant suppression of primary tumor growth (p < 0.001) by a SeMet-supplemented diet (3 mg kg^-1^ L-SeMet from Sabinsa Corporation, East Windsor, NJ) compared to a Se-deficient diet (< 0.01 mg kg^-1^ Se). Additionally, on the 16^th^ day after cancer cell inoculation, tumor volume was already affected by the Se status. In the present study, diets were introduced after tumor inoculation, while Chen et al. [45] initiated diets 3 months prior to 4T1 cell inoculation.

The results from another study performed by Song et al. [60] also corroborates with ours, since SeMet supplementation for 28 days could reduce tumor volume compared with PBS. Notable differences were observed only after the 19^th^ day of feeding. Mice in the former experiment received individual orally administered SeMet (gavage), while in the present study, Se was included in the feed. Differences in daily consumption of experimental diets by each animal may be a possible explanation for these small discrepancies. Although individual doses allow the exact determination of a dose-response effect relationship, daily injections in the stomach is a stressful event for long-term experiments involving nutrients. Although, the therapeutic intervention by Song et al. [60] occurred at an earlier stage of carcinogenesis and may have promoted different anticancer effects through different selenoproteins [42], their results were quite similar to ours. Conversely, in our experiment, the control group received Se-adequate feed instead of PBS. This fact highlights the possible influence of the food matrix, which can alter Se bioaccessibility and bioavailability, affecting, therefore, biological effects from dietary intake [73, 74].

The matrix of Brazil nuts is quite complex and, therefore, interferes with the activity of its Se compounds [75, 76]. According to Dumont et al. [76] the main compounds present in the matrix after proteolytic digestion of Brazil nuts are Se-(Cys)2 and Se-Met, the latter being the major compound, which is also supported by recent research [77]. Silva et al. [78] performed an in vitro bioaccessibility test and observed that only selenomethionine was found to be bioaccessible in Brazil nuts, corresponding to 74% of the total selenium present in the sample. This result is in agreement with others in the literature, which lalso showed this species as the most abundant in Brazil nuts, with concentrations ranging from 75% to 96% of the total concentration [34, 79]. The lack of a significant difference in the blood Se levels from animals given Se-Nuts compared to those of the control group may have two possible explanations: 1) Se provided by nuts could show poorer absorption than pure SeMet (since SeMet is reported to be the most bioavailable Se compound) [80]. 2) Considering that tumor volume was significantly lower in both Se-supplemented groups (than the control group), probably Se from Brazil nuts shows lower retention by the body tissues than Se from the SeMet group [81, 82]. Vitamin E can also be present in Brazil nuts [83], and its interaction with Se should be taken into consideration [84] since it can enhance antioxidant defense in organisms [85] parallel to Se.

Brazil nuts contain high levels of unsaturated fatty acids, both monounsaturated (MUFAs) and polyunsaturated (PUFAs) [34, 86], and changes in the activity of enzymes from the GPx family have been described during PUFA supplementation [87, 88]. Consumption of Brazil nuts by obese women for 8 weeks increased GPx-1 erythrocyte activity, but the association between GPx activity and erythrocyte Se concentration was not the same among different genotypes [89]. GPx belongs to a group of stress-related selenoproteins [90], and since GPx-1 allelic identity is associated with breast cancer development [91], decreased tumor growth was expected to be associated with increased hepatic GPx activity in the Se-Nuts group compared to the control group.

Tumor-bearing mice exhibited lower GPx activity in plasma in a previous study [4], and the activity of GPx in tissues is more sensitive to dietary Se deficiency than that of other selenoproteins [92, 93]. Therefore, we expected a difference between the Se-adequate and SeMet groups. Although hepatic GPx activity was similar among groups, the hepatic GPx activity in the control group (0.15 mg kg^-1^ Se) was similar to that found in a previous study [30].

In summary, organic Se-supplemented diets were effective in suppressing tumor growth. Additionally, Se supplemented from Brazil nuts did not improve the blood Se concentration as the SeMet diet did, although there were no differences in hepatic GPx-1 activity. This finding suggests that SeMet supplementation may not affect hepatic GPx-1 before improving blood selenoproteins, which involves mainly plasma SelP, extracellular GPx-3, erythrocytes GPx-1, as well as lower concentrations of SeMet, trimethylselenonium ion, and selenosugar in red blood cells [65, 66]. More research is needed to elucidate the specific mechanisms of Se compounds to develop therapeutic protocols. Although both Se-supplemented diets (SeMet and Se-Nuts) contained 1.4 mg/kg of total Se, only the SeMet group showed a higher blood Se concentration. Since hepatic GPx-1 activity did not respond to either Se-supplemented diet, evaluation of these parameters (blood Se concentration and hepatic GPx-1 activity) suggests that SeMet supplementation may not affect hepatic GPx-1 before improving blood selenoproteins.

## Conclusions

Selenium-rich Brazilian nuts and selenomethionine dietary supplementation, starting after detection of 4T1 palpable lesions, reduced tumor volume in mice in comparison to Se-adequate diet.

## Supporting information

S1 Checklist (PDF)

S1 Data (XLSX)
